# A New Hypothesis on the Etiology of Down Syndrome: The Role of Anti-Zona Pellucida Antibodies as an Age-Independent Factor

**DOI:** 10.3390/ijms27020991

**Published:** 2026-01-19

**Authors:** Giuseppe Noia, Tina Pasciuto, Francesco Ria, Alfredo Pontecorvi, Monica Sacco, Emanuela Teveroni, Maurizio Genuardi, Francesca Mauro, Paolo Spina, Emilia Spina, Giada Castagna, Daniela Visconti, Antonio Lanzone, Marco De Santis

**Affiliations:** 1 UOC Obstetrics and Obstetric Pathologies, Department of Woman and Child Health and Public Health, Fondazione Policlinico Universitario A. Gemelli IRCCS, 00136 Rome, Italy; giuseppe.noia@guest.policlinicogemelli.it (G.N.); giada.castagna.1@studenti.unipd.it (G.C.); daniela.visconti@policlinicogemelli.it (D.V.); antonio.lanzone@policlinicogemelli.it (A.L.); marco.desantis1@policlinicogemelli.it (M.D.S.); 2 “Il Cuore in una Goccia” Non-Profit Foundation, 00167 Rome, Italy; francescamauro@yahoo.it (F.M.); paol.spina@gmail.com (P.S.); emiliaspina77@gmail.com (E.S.); 3 Research Core Facility Data Collection G-STeP, Fondazione Policlinico Universitario A. Gemelli IRCCS, 00136 Rome, Italy; 4 Section of Hygiene, University Department of Life Sciences and Public Health, Università Cattolica del Sacro Cuore, 00168 Rome, Italy; 5 Department of Translational Medicine and Surgery, Section of General Pathology, Università Cattolica del Sacro Cuore, 00168 Rome, Italy; francesco.ria@policlinicogemelli.it; 6 Operative Unit of Internal Medicine, Endocrinology & Diabetes, Fondazione Policlinico Universitario A. Gemelli IRCCS, 00136 Rome, Italy; alfredo.pontecorvi@policlinicogemelli.it; 7 Department of Translational Medicine and Surgery, Facoltà Medicina e Chirurgia “Agostino Gemelli”, Università Cattolica del Sacro Cuore, 00168 Rome, Italy; monica.sacco@guest.policlinicogemelli.it; 8 Clinical Chemistry, Biochemistry and Molecular Biology Operations (UOC), Fondazione Policlinico Universitario A. Gemelli IRCCS, 00168 Rome, Italy; ema.teveroni@gmail.com; 9 Istituto Scientifico Internazionale “Paolo VI”, Fondazione Policlinico Universitario A. Gemelli IRCCS, 00136 Rome, Italy; 10 Genomic Medicine, UOC Medical Genetics Department of Life Sciences and Public Health, Università Cattolica del Sacro Cuore, 00168 Rome, Italy; maurizio.genuardi@policlinicogemelli.it; 11 Section of Gynaecology and Obstetrics, University Department of Life Sciences and Public Health, Università Cattolica del Sacro Cuore, 00168 Rome, Italy

**Keywords:** anti-zona pellucida antibodies, Down syndrome, trisomy 21, anti-thyroid antibodies, pregnancy

## Abstract

Down Syndrome (DS) is the most common chromosomal abnormality characterized by neurodevelopmental impairment. Apart from maternal age, its risk factors remain poorly understood. This prospective case-control study aimed to evaluate the role of maternal anti-zona pellucida (ZP) antibodies (Ab) and anti-thyroid-Ab in predicting DS. Correlations of anti-ZP-Ab and anti-thyroid-Ab with maternal age were also assessed. Anti-ZP-Ab were measured after childbirth using ELISA. Anti-thyroid peroxidase (aTPO) and anti-thyroglobulin (aTgII) antibodies were also analysed with the Allelica IM platform. Statistical analyses included receiver operating characteristic curve assessment, expressed as area under the curve (AUC) and linear regression modeling. Between September 2020 and October 2022, 58 women were enrolled. Anti-ZP-Ab levels were significantly higher in women with DS pregnancy with an odds ratio adjusted for maternal age of 71.52 (95% CI: 7.05–725.18) and an excellent predictive performance (AUC = 0.94; 95% CI: 0.88–1.00). For optical density levels > 1, the accuracy was 89.7% (95% CI: 78.2–100.0). No statistically significant differences were observed for aTPO and aTgII. Neither Anti-ZP-Ab nor anti-thyroid antibodies increased with age. These findings suggest that Anti-ZP-Ab are strongly associated with DS risk, suggesting a potential age-independent autoimmune contribution to trisomy 21. Their evaluation may support preconception counseling, especially for women aged > 35 years. Future studies could clarify causality and define the role of maternal autoimmunity in DS etiology.

## 1. Introduction

Down Syndrome (DS) is the most common chromosomal abnormality, characterized by a neurodevelopmental disorder with a prevalence of about 1/700–1/1000 live births [1,2,3]. Its frequency varies according to maternal age distribution, access to prenatal screenings, and pregnancy termination practices. DS most commonly results from free trisomy 21 (T21), which arises from meiotic nondisjunction during oogenesis. Maternal age is the strongest known risk factor for T21 and other aneuploidies related to chromosome nondisjunction during maternal meiosis, particularly the first meiotic division. In fact, the risk of a T21 birth rises from about 3% for younger women in their 20s to 30% in older women in their 40s [4,5,6]. Despite the well-established epidemiological association, the biological mechanisms linking maternal aging to meiotic errors remain incompletely understood [7,8,9]. Proposed contributors include age-related deterioration of cohesin complexes, altered recombination patterns, and impaired spindle assembly checkpoint function [5,6].

Beyond maternal age, several genetic, epigenetic, and environmental factors have been implicated in modulating DS risk. Variants in gene involved in folate metabolism—particularly *MTHFR* c.677C>T and c.66A>G—have been associated with altered methylation pathways and increased susceptibility to meiotic nondisjunction [10]. Additionally, influences include maternal obesity, smoking, and metabolic alterations that affect serum biomarkers and oocyte quality. However, these factors explain only a fraction of DS cases, suggesting that additional mechanisms may contribute to the etiology of T21 pregnancies [10,11,12].

A growing body of evidence indicates that autoimmune processes targeting ovarian structures may impair folliculogenesis and oocyte competence. Experimental studies have shown that immunization with zona pellucida (ZP) proteins induces ovarian inflammation, follicular depletion, and infertility in animal models [13,14]. Anti-ZP antibodies have been detected in women with premature ovarian failure and diminished ovarian reserve, supporting a role for autoimmune ovarian dysfunction in human reproductive disorders [15]. These findings suggest that immune-mediated damage to the oocyte or its surrounding structures may compromise meiotic integrity.

Interestingly, epidemiological observations have reported that mothers of children with DS tend to experience menopause at a younger age compared with mothers without DS offspring [16]. Moreover, women who delivered a child with DS at a young age often exhibit reduced ovarian reserve, including lower anti-Müllerian hormone (AMH) levels [17,18]. These data raise the possibility that intrinsic or immune-mediated ovarian dysfunction may predispose to meiotic errors independently of chronological age.

Given the established role of immune factors in ovarian aging and oocyte quality [19,20,21,22], and the evidence linking anti-ZP antibodies to impaired follicular function, we hypothesized that anti-ZP autoimmunity may contribute to the etiology of T21 pregnancies. The present study was therefore designed to investigate the association between anti-zona pellucida antibodies and the occurrence of trisomy 21, exploring whether autoimmune ovarian factors may represent an age-independent risk mechanism for DS.

## 2. Results

Between September 2020 and October 2022, 58 women (29 cases and 29 controls) were prospectively enrolled. Gestational and clinical characteristics of the 29 dyads—mother and DS child—are reported in Table 1. The mean maternal age for DS pregnancies was 37.1 years (SD: 5.2) with a median gestational age of 38.6 weeks (SD: 3.1). Delivery was spontaneous in 10/19 cases (52.6%). At birth, the mean weight was 2719.3 g.

Clinical and serological characteristics of cases and controls are described in Table 2. No statistically significant differences in terms of age at menarche and at serological evaluation, autoimmune disease, other concomitant pathologies and drug assumptions, including current or past hormonal therapies, were observed. The median number of parities was 3 (min–max: 1–8) and 2 (min–max: 1–3) for cases and controls (*p* = 0.002), respectively.

Overall, the median age at serological evaluation was 40.4 years (SD: +5.7) and no statistically significant differences were found in the distribution of the after child anti-thyroid peroxidase (aTPO) and anti-thyroglobulin (aTgII) antibodies (*p* = 0.81 and *p* = 0.24 respectively), while the anti-ZP antibodies were significantly higher in the case group (*p* < 0.001).

**Figure 1:**
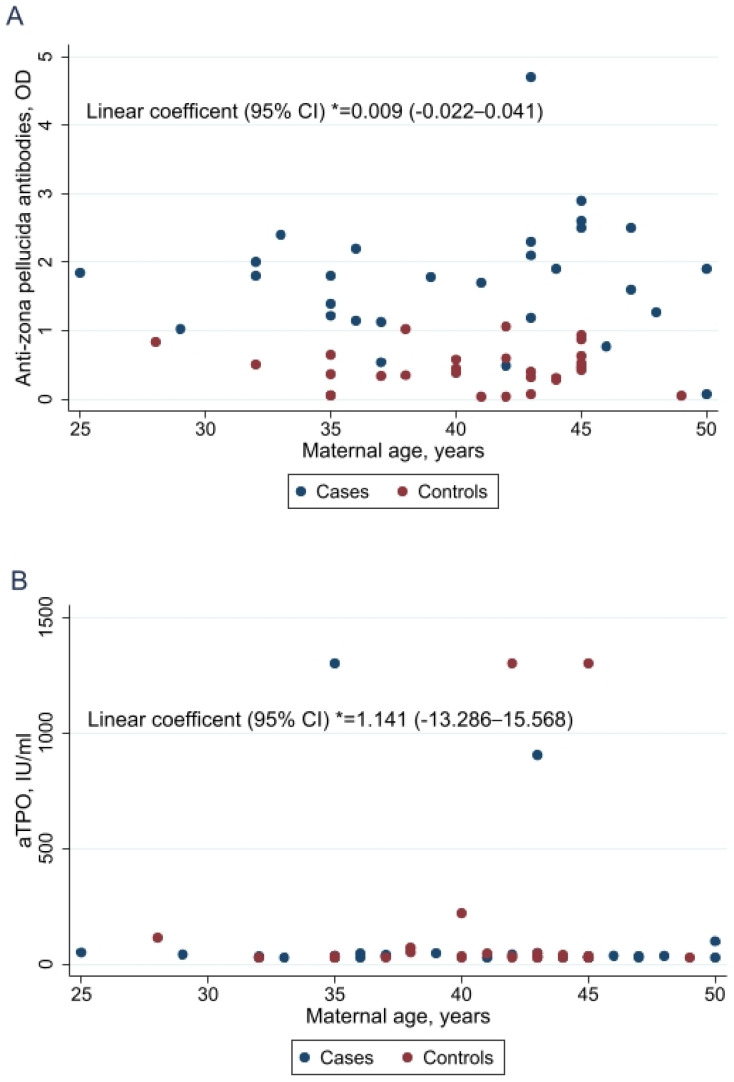

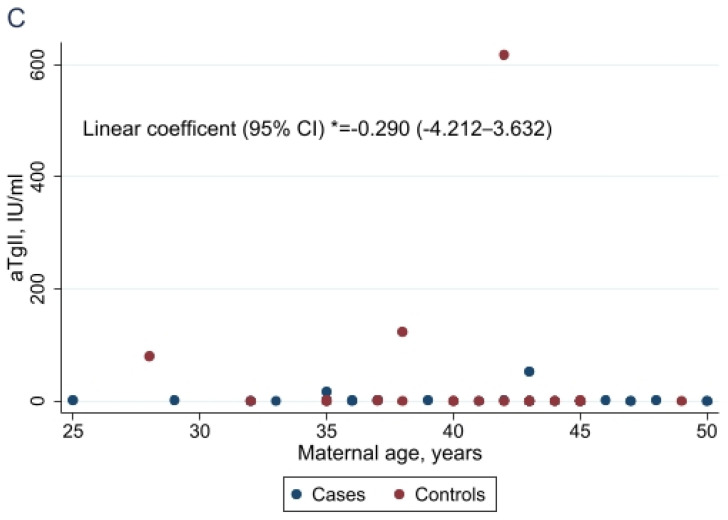
Scatter plot of maternal age and anti-zona pellucida antibodies (**A**), anti-Thyroid Peroxidase antibodies (**B**) and anti-Thyroglobulin antibodies (**C**). * Linear coefficient refers to the coefficient in linear regression analysis adjusting for Down syndrome pregnancy. Confidence interval (CI). IU: International Unit. OD: Optical Density.

The value of anti-ZP as well anti-thyroid antibodies did not increase with age. These results were investigated both analysing separately cases and controls and analysing the overall correlation of maternal age and anti-ZP or anti-thyroid antibodies adjusting for DS pregnancy (Figure 1).

**Figure 2:**
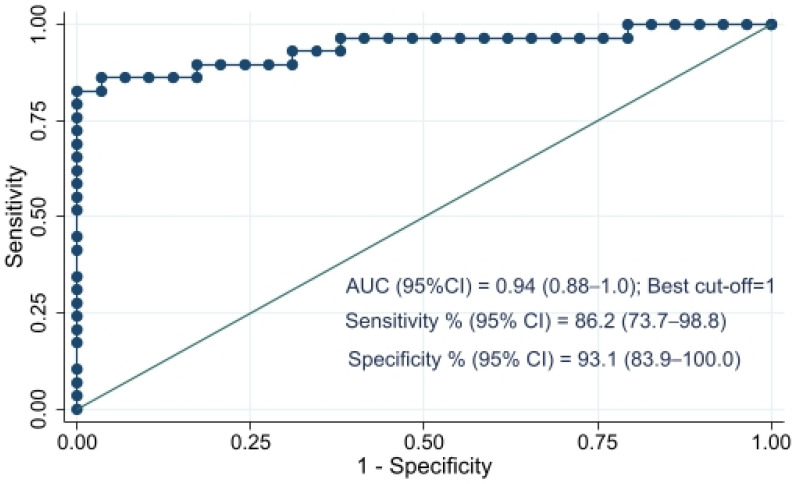
Receiver Operating Characteristic curve of anti-zona pellucida antibodies in predicting Down syndrome pregnancy Results, including Area Under the Curve (AUC) are shown with 95% Confidence Intervals.

ROC analysis, shown in Figure 2, shows that the performance of anti-ZP antibodies in DS pregnancy prediction has an AUC of 0.94 (95% CI: 0.88–1.00). The sensitivity and specificity associated with the best cutoff of 1 OD retrieved through Liu’s method are 86.2% (95% CI: 73.7–98.8) and 93.1% (95% CI: 83.9–100.0), respectively, with an accuracy of 89.7% (78.2–100.0). The associated OR adjusted for maternal age was 71.52 (95% CI: 7.05–725.18).

Since the cut-off value of 2.1 OD is commonly used in the literature, we also calculated the frequency of DS pregnancies using this threshold. We found that 34.5% of women with a DS pregnancy had anti-ZP antibody values higher than 2.1 OD, whereas none of the controls exceeded this value. (Table 2).

## 3. Discussion

In this prospective case-control study, we found that anti-ZP antibodies are significantly associated with an increased risk of DS pregnancy. To our knowledge, this is the first prospective study investigating the role of anti-ZP antibodies in predicting the risk of DS pregnancy.

In our series many DS pregnancies occurred in women aged > 35 years, as expected [23]. Adjusting our results for maternal age, anti-ZP antibodies emerged as an additional independent risk factor for trisomy 21. Moreover, we found that values higher than 1 OD can predict DS pregnancy with an excellent AUC. This cut-off was chosen to maximize sensitivity and specificity and is tailored for DS pregnancy prediction.

### 3.1. Anti-ZP Antibodies as an Age-Independent Risk Factor

Maternal age is the most established determinant of meiotic nondisjunction, with error rates increasing markedly as women age [24,25]. However, several studies have shown that maternal age alone does not fully explain the occurrence of trisomy 21. Sherman et al. (1994) demonstrated that reduced recombination on chromosome 21q is linked to nondisjunction events, particularly during the meiosis I, and that these recombination patterns are not solely age-dependent [26]. Indeed, most trisomy 21 cases originate from maternal meiotic errors (92% maternal vs. 4.9% of paternal origin), with the majority occurring during meiosis I [27].

Recombination plays a critical role in ensuring proper chromosome orientation on the meiotic spindle. Reduced or absent recombination increases the risk of meiotic nondisjunction (ND). Importantly, the overall reduction in recombination frequency appears to be independent of maternal age, while the location of chiasmata influences whether errors occur in meiosis I or II [28,29,30]. Distal single exchanges are more common in younger women with meiosis I errors, whereas proximal chiasmata are more typical in older women with meiosis II errors. These findings support the existence of both age-dependent and age-independent pathways leading to ND.

Our results align with this concept, suggesting that anti-ZP antibodies may represent an additional, age-independent mechanism contributing to meiotic errors.

### 3.2. Autoimmunity as a Contributor to Meiotic Dysfunction

Given the multifactorial nature of nondisjunction, autoimmune mechanisms have been proposed as potential contributors to DS risk. Charkiewicz et al. (2016) identified significant differences in the expression of over 200 autoantibodies in women with DS pregnancies, including antibodies targeting proteins involved in oocyte maturation and chromosomal segregation [31]. Similarly, Mikwar et al. (2020) highlighted several pathways implicated in age-related aneuploidy—recombination failure, cohesin impairment, spindle assembly checkpoint dysfunction, epigenetic dysregulation, and mitochondrial defects—some of which may be influenced by autoantibodies [32]. Our findings reinforce the hypothesis that autoimmune dysregulation may impair oocyte quality and increase the risk of meiotic errors, independently of maternal age.

### 3.3. Biological Plausibility: Anti-ZP Antibodies and Ovarian Dysfunction

Anti-ZP antibodies have long been associated with infertility [33,34,35,36,37] and premature ovarian failure (POF). The broader concept that endocrine autoimmunity may contribute to ovarian dysfunction is supported by evidence of prematurely diminished ovarian reserve and by the observed association between androgen production and immune system activation. This relationship has been proposed as indirect evidence for a functional adrenal–ovarian autoimmune axis in women [38].

Experimental studies in mice demonstrated that exposure to anti-ZP antibodies impairs folliculogenesis, reducing follicle diameter, antral formation, mucification, oocyte maturation, and fertilization rates. Morphological changes included thinner zona pellucida and fewer granulosa cell microvilli penetrating the ZP, disrupting oocyte–granulosa communication [39,40,41]. These findings suggest that anti-ZP antibodies can damage follicle development and potentially contribute to meiotic nondisjunction.

Additional evidence comes from studies of thyroid autoimmunity. In women with diminished ovarian reserve, thyroid autoantibodies have been associated with poorer embryonic quality, suggesting that autoimmune activation—even when not directed specifically against ovarian structures—can negatively affect female fertility [42,43,44,45,46,47,48].

Cross-reactivity of anti-ZP antibodies with thyroid microsomal components has also been reported. Kelkar et al. (2005) [15] found that patients with POF often had anti-ovary antibodies, many of which cross-reacted with ZP and thyroid tissue. In humans, a significant proportion of women with anti-ZP antibodies also had anti-thyroid antibodies, suggesting a link between ovarian and thyroid autoimmunity [15]. This is particularly relevant given that DS itself is strongly associated with autoimmune thyroid disease. Reduced expression of the autoimmune regulator (AIRE) gene in DS thymus tissue has been linked to increased susceptibility to thyroid autoimmunity [49].

Taken together, these data support a broader framework in which autoimmune processes may influence ovarian function, oocyte competence, and ultimately the risk of trisomy 21. Anti-ZP antibodies are generally understood to arise as part of an autoimmune response directed against zona pellucida glycoproteins. Such antibodies may develop following exposure to ovarian antigens due to follicular disruption, ovarian inflammation, or other immune-modulating conditions. However, the precise trigger for their production in individual patients cannot be determined from our dataset.

Finally, our study was not designed to identify the initial immunological source of anti-ZP antibody production. Future mechanistic research will be required to clarify the pathways leading to their development and to define their role in ovarian dysfunction.

### 3.4. Strengths and Limitations

The strengths of this study include its prospective design, rigorous laboratory methodology, and the strong predictive performance of anti-ZP antibody levels. However, several limitations must be acknowledged. First, anti-ZP antibodies were measured postpartum, preventing us from determining whether they were present before conception or developed during or after pregnancy. Future studies incorporating pre-pregnancy sampling will be essential to establish causality. Second, because cases and controls were matched by age to ensure comparability, we could not directly evaluate the interaction between maternal age and anti-ZP antibodies.

Nevertheless, our analyses adjusted for maternal age, and the association between anti-ZP antibodies and DS remained significant.

### 3.5. Implications and Future Directions

For many years, maternal age has been considered the primary determinant of trisomy 21. Our findings suggest that additional factors—particularly autoimmune dysregulation involving the development of anti-ZP antibodies—may contribute to the etiology of DS, potentially playing a more prominent role in younger women. If confirmed, this hypothesis could have important implications for preconception counseling and risk assessment.

Further research is needed to clarify the temporal relationship between anti-ZP antibodies and pregnancy, to explore the mechanistic pathways linking autoimmunity to meiotic errors, and to determine whether anti-ZP antibodies could serve as a biomarker for DS risk in clinical practice.

## 4. Materials and Methods

### 4.1. Study Design and Ethical Approval

This prospective case-control study was performed at Fondazione Policlinico Universitario A. Gemelli–IRCCS. The study protocol was approved by the local Ethical Committee (Prot. N. 6826/20 ID 3012), and written informed consent was obtained from all participants prior to enrolment.

### 4.2. Eligibility Criteria

The study aimed to evaluate the prevalence of anti-ZP antibodies in women who had delivered a child with Down syndrome (cases) compared with women with no history of Down syndrome pregnancies (controls). Participants were considered eligible if they were aged > 20 years old, not pregnant at the time of enrolment, and had no major metabolic or vascular diseases including diabetes, hypertension, or antiphospholipid antibodies syndrome. Additional exclusion criteria for controls were any previous pregnancy affected by genetic abnormalities and/or congenital malformations. Cases and controls were matched by age.

Secondary endpoints were: (a) the association of anti-thyroid antibodies levels and DS pregnancy; (b) the correlation of anti-ZP and anti-thyroid antibodies and age.

### 4.3. Data Collection

Study data were prospectively collected and managed in compliance with the General Data Protection Regulation (GDPR) using REDCap electronic data capture tools hosted at Fondazione Policlinico Universitario A. Gemelli-IRCCS (https://redcap-irccs.policlinicogemelli.it/ (accessed on 20 November 2022)) [50].

Authenticated and restricted access to the platform was allowed only for people officially registered as study investigators or authorized data managers.

### 4.4. Sample Collection and Processing

For each participant, a venous blood sample was obtained for measurements of anti-ZP antibodies, thyroid hormones, and anti-thyroid antibodies.

Blood samples were allowed to clot for 24 h at +4–+8 °C. Serum was obtained by centrifugation at 1500× *g* for 10 min, aliquoted into 0.5 mL fractions, and stored at −20 °C until analysis. Samples were thawed once at room temperature and used within 2 h. Repeated freeze-thaw cycles were avoided due to loss in antibody activity.

### 4.5. Laboratory Analyses

#### 4.5.1. Measurement of Human Anti-Zona Pellucida Antibodies

Human anti-ZP IgG antibodies (azp-Ab) were measured using a commercial ELISA kit (Wuhan Huamei Biotech Co., Ltd.—operating as CUSABIO—Wuhan, Hubei Province, China), following the manufacturer’s instructions. The kit used was catalog number CSB-E09082h (lot numbers C0604050604 and C0633130638). Detection was performed using an anti-human IgG secondary antibody, conjugated with Horseradish Peroxidase (HRP), reacting with the chromogenic substrate tetramethylbenzidine (TMB). Optical density (OD) was measured with a microplate reader set to 450 nm. Tests were performed in double. The Intra-assay and inter-assay precision showed a coefficient of variation (CV) <15%. According to the manufacturer’s instructions, results were expressed as OD sample/OD negative control (provided with the kit). In our study, the mean ratio ± standard deviation obtained from control and cases samples were respectively 0.44 ± 0.28 and 2.01 ± 0.67. According to the manufacturer’s cut-off, a sample was considered positive for azp-Ab when the ratio OD sample/OD negative was ≥2.1.

#### 4.5.2. Measurement of Anti-Thyroid Antibodies

Anti-thyroid peroxidase (aTPO) and anti-thyroglobulin (aTgII) antibodies were centrally measured using the Atellica IM analyser (Siemens Healthineers, Erlangen, Germany) at the high automation CoreLab of Fondazione Policlinico Universitario A. Gemelli-IRCCS.

##### aTgII Assay

The aTgII method is a competitive bridge immunoassay based on chemiluminescence detection. This method uses two different types of human thyroglobulin. Briefly, the reagent containing human thyroglobulin labelled with acridinium ester was incubated with the serum sample test and, subsequently, with the solid phase containing a biotinylated human thyroglobulin bound to paramagnetic beads which, in turn, are coupled to streptavidin. The aTgII antibodies present in the serum sample bind both the acridinium esterified-thyroglobulin in the reagent and the biotinylated-thyroglobulin in the solid phase, forming a bridge that allows chemiluminescent detection. The aTgII antibodies title was expressed in IU/mL and based on a specific standard calibration curve. The aTgII cut-off value ≥ 4.5 IU/mL indicates thyroid autoimmune disorders. The sensitivity is 98.5% (95% Confidence Interval (CI): 91.7–100%); while the specificity is 94.8% (95% CI: 90.3–97.6%). The measurement range in which the method is linear, is 1.3–1000 IU/mL with LoD < 1.3 IU/mL.

##### aTPO Assay

Similarly, the aTPO Atellica IM method is a competitive immunoassay based on chemiluminescence detection. This method uses a mouse monoclonal aTPO antibody bound to paramagnetic beads in the solid phase which competes with the possible aTPO antibody in the serum sample test for binding with a limited quantity of human TPO complexed with a mouse monoclonal aTPO acridinium ester-labelled antibody.

The aTPO antibodies title was expressed in U/mL and based on a specific standard calibration curve. The measurement range in which the method is linear is 28–1000 IU/mL in serum and plasma samples with LoD < 28 U/mL.

### 4.6. Statistical Analyses

Assuming a case control design with a 1: 1 ratio, type 1 error α = 0.05, type 2 error β = 0.20 (study power 80%) and assuming that in the general population the prevalence of anti-ZP antibodies is about 20% [36,41], an enrolment of 58 total patients (29 cases and 29 controls) was estimated in order to observe a prevalence of 55% of anti-ZP antibodies in women with DS children. The sample was calculated using a two-tailed test using the STATA software version 13.

Study characteristics were presented in terms of absolute frequency (percentage relative frequency) for qualitative variables and in terms of mean (standard deviation) and median (min–max) in the case of normal and not normal distribution of quantitative variables respectively. Shapiro-Wilks’ test was used to assess the normality of continuous variables distribution. Cases and controls comparisons were made through two-sided Pearson’s χ^2^ or Fisher’s exact test and Student’s *t* test or Mann-Whitney’s test as appropriate.

The correlation of maternal age with anti-ZP antibodies, as well with anti-thyroid antibodies (aTPO and aTgII) was assessed separately in cases and controls, and overall, through linear regression analysis adjusting for DS pregnancy.

Receiver–operating characteristic (ROC) analysis was applied to evaluate the ability of anti-ZP antibodies to predict DS pregnancy in terms of area under the curve (AUC), as well as to determine the best cut-off value for this prediction. Best cut-off was detected according to Liu’s method which defines the best cut-point as the point maximizing the product of sensitivity and specificity index [51]. Sensitivity, specificity and accuracy were also calculated. All performance parameters were provided with 95% Confidence Intervals (CIs). AUC was classified as fair in case of values between 0.70–0.80, good for >0.8 AUC < 0.9 and excellent for values > 0.9 [52].

The effect of anti-ZP antibodies in predicting a DS pregnancy was evaluated through logistic regression analysis adjusting for maternal age and was presented in terms of Odds Ratio (OR).

All estimates were provided with 95% CI. No imputation was performed for missing data and the level of significance was set at *p* = 0.05. Statistical analysis was performed by an experienced biostatistician (TP) using STATA software (STATA/BE 17.0 for Windows, Stata Corp LP, College Station, TX 77845, USA).

## 5. Conclusions

In this prospective case–control study, we demonstrated that anti-ZP antibodies are significantly associated with an increased risk of DS pregnancy. This association remained robust even after adjusting for maternal age, suggesting that anti-ZP antibodies may represent an age-independent risk factor for T21. We also identified that anti-ZP antibody levels above 1.0 OD predict DS pregnancy with excellent performance, indicating potential clinical utility for preconception or early-pregnancy risk assessment.

These findings support the broader hypothesis that autoimmune mechanisms may contribute to meiotic errors, complementing the well-established role of maternal age. The biological plausibility of this association is reinforced by previous evidence linking anti-ZP antibodies to impaired folliculogenesis, diminished ovarian reserve, and premature ovarian failure—all conditions that may compromise oocyte quality and increase susceptibility to nondisjunction.

While our study provides novel insights, several questions remain open. Because anti-ZP antibodies were measured postpartum, the temporal relationship between antibody production and conception cannot be fully established. Future longitudinal studies with pre-pregnancy sampling will be essential to clarify causality and to determine whether anti-ZP antibodies could serve as a reliable biomarker for DS risk. Additional research is also needed to explore the mechanistic pathways through which autoimmune dysregulation may influence meiotic competence.

Overall, our results suggest that maternal autoimmunity—particularly the presence of anti-ZP antibodies—may play a previously underappreciated role in the etiology of trisomy 21. If confirmed, this hypothesis could have important implications for preconception counseling, reproductive planning, and personalized risk assessment, especially for women who may be at risk independently of age.

## Figures and Tables

**Table 1:** Gestational and clinical characteristics of the dyad mother and Down syndrome child.

Characteristics	Cases n = 29
Mother	
Age at DS pregnancy	37.1 ± 5.2
Previous parities	1 (0–6)
Previous spontaneous abortion before DS pregnancy	0 (0–4)
Previous pregnancies with congenital anomalies	5/25 (20.0)
Gestational age at DS delivery, weeks	38.6 ± 3.1
Type of delivery	
Caesarean	10/19 (52.6)
Natural	9/19 (47.4)
Down syndrome child	
Gender	
Female	15/28 (53.6)
Male	13/28 (46.4)
ABO blood groups combined with Rh factor status	
0−	1/19 (5.3)
0+	8/19 (42.1)
A−	1/19 (5.3)
A+	7/19 (36.8)
B−	1/19 (5.3)
B+	1/19 (5.3)
Weight at birth, gr	2719.3 ± 777.1
Other associated pathologies at birth	21/28 (75.0)
Breastfeeding	8/22 (36.4)

Categorical variables are expressed as n (%) or n/number of available data (%). Normally-distributed continuous variables are presented as mean ± standard deviation, while non-normally distributed variables are presented as median and range (min–max). DS: Down Syndrome. ABO system: 0 indicates absence of A and B antigens; A indicates presence of A antigen; B indicates presence of B antigen. Rh factor: “+” denotes Rh-positive (Rh antigen present), and “−” denotes Rh-negative (Rh antigen absent).

**Table 2:** Clinical and serological characteristics of women who have had Down children (cases) and who have had not Down syndrome pregnancies (controls).

Characteristics	All Patients n = 58	Cases n = 29	Controls n = 29	*p*-Value
Age of menarche, years	12.6 ± 1.1	12.5 ± 0.7	12.7 ± 1.3	0.53
Age at serological evaluation, years				
Continuous value	40.4 ± 5.7	40.1 ± 6.5	40.7 ± 4.8	0.68
Dichotomized value				0.42
<35 years	7/58 (12.1)	5/29 (17.2)	2/29 (6.9)	
≥35 years	51/58 (87.9)	24/29 (82.8)	27/29 (93.1)	
Total parities	2 (1–8)	3 (1–8)	2 (1–3)	**0.002**
Autoimmune diseases				0.74
No	46/58 (79.3)	22/29 (75.9)	24/29 (82.8)	
Yes	12/58 (20.7)	7/29 (24.1)	5/29 (17.2)	
Other concomitant diseases				0.65
No	36/57 (63.2)	17/29 (58.6)	19/28 (67.9)	
Yes	21/57 (36.8)	12/29 (41.4)	9/28 (32.1)	
Drug therapies				0.39
No	40/58 (69.0)	18/29 (62.1)	22/29 (75.9)	
Yes	18/58 (31.0)	11/29 (37.9)	7/29 (24.1)	
Current and past hormonal therapy				1
No	47/57 (82.5)	24/29 (82.8)	23/28 (82.1)	
Yes	10/57 (17.5)	5/29 (17.2)	5/28 (17.9)	
Maternal ABO blood group				0.92
0	19/47 (40.4)	9/24 (37.5)	10/23 (43.5)	
A	18/47 (38.3)	10/24 (41.7)	8/23 (34.8)	
AB	5/47 (10.6)	3/24 (12.5)	2/23 (8.7)	
B	5/47 (10.6)	2/24 (8.3)	3/23 (13.0)	
Rh antigen				1
Positive	41/55 (74.5)	21/28 (75.0)	20/27 (74.1)	
Negative	14/55 (25.5)	7/28 (25.0)	7/27 (25.9)	
aTPO, UI/mL				
Continuous value	15.6 ± 83	2.8 ± 10	28.3 ± 116.5	0.81
Dichotomized according to literature				0.71
≤60	50 (86.2)	26 (89.7)	24 (82.8)	
>60	8 (13.8)	3 (10.3)	5 (17.2)	
aTgII, IU/mL				
Continuous value	120.4 ± 301.6	110.6 ± 280	130.2 ± 326.5	0.24
Dichotomized according to literature				0.61
≤45	54 (93.1)	28 (96.6)	26 (89.7)	
>45	4 (6.9)	1 (3.4)	3 (10.3)	
Anti-zona pellucida antibodies, OD				
Continuous value	1.1 ± 0.9	1.8 ± 0.9	0.5 ± 0.3	**<0.0001**
Dichotomized according to literature				**0.001**
<2.1	48 (82.8)	19 (65.5)	29 (100.0)	
≥2.1	10 (17.2)	10 (34.5)	0 (0.0)	
Dichotomized according to ROC analysis				**<0.0001**
≤1	31 (53.4)	4 (13.8)	27 (93.1)	
>1	27 (46.6)	25 (86.2)	2 (6.9)	

Categorical variables are expressed as n (%) or n/number of available data (%). Normally-distributed continuous variables are presented as mean ± standard deviation, while non-normally distributed variables are presented as median and range (min–max). Shapiro-Wilk test was used to test normality. Comparisons between cases and controls were performed using two-side Pearson’s Chi-Square test or Fisher’s exact test for categorical variables, and Student’s *t*-test and Mann-Whitney U test for continuous variables, as appropriate. A fixed significance level of 5% was adopted; *p*-values in bold indicate statistically significant differences. IU: International Unit. ABO system: 0 indicates absence of A and B antigens; A indicates presence of A antigen; B indicates presence of B antigen. aTPO: Anti-Thyroid Peroxidase antibodies. aTgII: Anti-Thyroglobulin antibodies. OD: Optical Density. ROC: Receiver Operating Characteristic curve.

## Data Availability

Due to privacy restrictions, the data are not publicly available but may be obtained from the corresponding author upon reasonable request.

## References

[1] Mai C.T., Isenburg J.L., Canfield M.A., Meyer R.E., Correa A., Alverson C.J., Lupo P.J., Riehle-Colarusso T., Cho S.J., Aggarwal D. (2019). National population-based estimates for major birth defects, 2010–2014. Birth Defects Res..

[2] Loane M., Morris J.K., Addor M.C., Arriola L., Budd J., Doray B., Garne E., Gatt M., Haeusler M., Khoshnood B. (2018). Twenty-year trends in the prevalence of Down syndrome and other trisomies in Europe: 1990–2014. Eur. J. Hum. Genet..

[3] de Graaf G., Buckley F., Skotko B.G. (2015). Estimates of the live births, natural losses, and elective terminations with Down syndrome in the United States. Am. J. Med. Genet. Part A.

[4] Hassold T., Hunt P. (2009). Maternal age and chromosomally abnormal pregnancies: What we know and what we wish we knew. Curr. Opin. Pediatr..

[5] Nagaoka S.I., Hassold T.J., Hunt P.A. (2012). Human aneuploidy: Mechanisms and new insights. Nat. Rev. Genet..

[6] Gruhn J.R., Zielinska A.P., Shukla V., Blanshard R., Capalbo A., Cimadomo D., Nikiforov D., Chan A.C.-H., Newnham L.J., Vogel I. (2019). Chromosome errors in human eggs shape natural fertility over reproductive life span. Science.

[7] Eichenlaub-Ritter U., Staubach N., Trapphoff T. (2010). Chromosomal and cytoplasmic context determines predisposition to maternal age-related aneuploidy: Brief overview and update on MCAK in mammalian oocytes. Biochem. Soc. Trans..

[8] Hunt P., Hassold T. (2010). Female meiosis: Coming unglued with age. Curr. Biol..

[9] Warburton D. (2005). Biological aging and the etiology of aneuploidy. Cytogenet. Genome Res..

[10] Coppedè F. (2015). The genetics of folate metabolism and maternal risk of Down syndrome. Front. Genet..

[11] Deng W.Q., Cawte N., Campbell N., Azab S.M., de Souza R.J., Lamri A., Morrison K.M., Atkinson S.A., Subbarao P., Turvey S.E. (2024). Maternal smoking DNA methylation risk score associated with health outcomes in offspring of European and South Asian ancestry. eLife.

[12] Rana M.S., Parvin M.M., Islam M.T. (2025). The Multifactorial Causes of Down Syndrome During Pregnancy: A Narrative Review of Genetic, Environmental, and Maternal Influences. Health Sci. Rep..

[13] Lloyd M.L., Papadimitriou J.M., O’Leary S., Robertson S.A., Shellam G.R. (2010). Immunoglobulin to zona pellucida 3 mediates ovarian damage and infertility after contraceptive vaccination in mice. J. Autoimmun..

[14] Monniaux D., Clément F., Dalbiès-Tran R., Estienne A., Fabre S., Mansanet C., Monget P. (2014). The ovarian reserve of primordial follicles and the dynamic reserve of antral growing follicles: What is the link?. Reproduction.

[15] Kelkar R.L., Meherji P.K., Kadam S.S., Gupta S.K., Nandedkar T.D. (2005). Circulating anti-zona pellucida antibodies in women with premature ovarian failure. J. Reprod. Immunol..

[16] van der Stroom E.M., König T.E., van Dulmen-den Broeder E., Elzinga W.S., van Montfrans J.M., Haadsma M.L., Lambalk C.B. (2011). Early menopause in mothers of children with Down syndrome?. Fertil. Steril..

[17] van Montfrans J.M., Dorland M., Oosterhuis G.J.E., van Vugt J.M.G., Rekers-Mombarg L.T.M., Lambalk C.B. (1999). Increased concentrations of follicle stimulating hormone in mothers of children with Down syndrome. Lancet.

[18] Kline J.K., Kinney A.M., Levin B., Kelly A.C., Ferin M., Warburton D. (2011). Trisomic pregnancy and elevated FSH: Implications for the oocyte pool hypothesis. Hum. Reprod..

[19] Wallace W.H., Kelsey T.W. (2010). Human ovarian reserve from conception to the menopause. PLoS ONE.

[20] Hummitzsch K., Irving-Rodgers H.F., Hatzirodos N., Bonner W., Sabatier L., Reinhardt D.P., Sado Y., Ninomiya Y., Wilhelm D., Rodgers R.J. (2013). A new model of development of the mammalian ovary and follicles. PLoS ONE.

[21] Kerr J.B., Myers M., Anderson R.A. (2013). The dynamics of the primordial follicle reserve. Reproduction.

[22] Broekmans F.J., Faddy M.J., Scheffer G., te Velde E.R. (2004). Antral follicle counts are related to age at natural fertility loss and age at menopause. Menopause.

[23] Blanco-Montaño A., Ramos-Arenas M., Yerena-Echevarría B.A., Miranda-Santizo L.D., Ríos-Celis A.L., Dorantes-Gómez A.T., Morato-Rangel A.J., Meza-Hernández J.A., Acosta-Saldívar E.D., Aguilar-Castillo C.D. (2023). Factores de riesgo en el origen del síndrome de Down [Risk factors in the origin of Down syndrome]. Rev. Med. Inst. Mex. Seguro Soc..

[24] Eichenlaub-Ritter U., Chandley A.C., Gosden R.G. (1988). The CBA mouse as a model for age-related aneuploidy in man: Studies of oocyte maturation, spindle formation and chromosome alignment during meiosis. Chromosoma.

[25] Brook J.D., Gosden R.G., Chandley A.C. (1984). Maternal ageing and aneuploid embryos--evidence from the mouse that biological and not chronological age is the important influence. Hum. Genet..

[26] Sherman S.L., Petersen M.B., Freeman S.B., Hersey J., Pettay D., Taft L., Frantzen M., Mikkelsen M., Hassold T.J. (1994). Non-disjunction of chromosome 21 in maternal meiosis I: Evidence for a maternal age-dependent mechanism involving reduced recombination. Hum. Mol. Genet..

[27] Allen E.G., Freeman S.B., Druschel C., Hobbs C.A., O’Leary L.A., Romitti P.A., Royle M.H., Torfs C.P., Sherman S.L. (2009). Maternal age and risk for trisomy 21 assessed by the origin of chromosome nondisjunction: A report from the Atlanta and National Down Syndrome Projects. Hum. Genet..

[28] Lamb N.E., Freeman S.B., Savage-Austin A., Pettay D., Taft L. (1996). Susceptible chiasma configurations of chromosome 21 predispose to nondisjunction in both maternal meiosis I and meiosis II. Nat. Genet..

[29] Oliver T.R., Feingold E., Yu K., Cheung V., Tinker S., Yadav-Shah M., Masse N., Sherman S.L. (2008). New insight into Human Nondisjunction of Chromosome 21 in Oocyte. PLoS Genet..

[30] Ghosh S., Feingold E., Dey K. (2009). Etiology of Down Syndrome: Evidence for Consistent Association among Altered Meiotic Recombination, Nondisjunction and Maternal Age Across Populations. Am. J. Med. Genet..

[31] Charkiewicz K., Zbucka-Kretowska M., Goscik J., Wolczynski S., Lemancewicz A., Laudanski P. (2016). Brief Communication: Maternal Plasma Autoantibodies Screening in Fetal Down Syndrome. J. Immunol. Res..

[32] Mikwar M., MacFarlane A.J., Marchetti F. (2020). Mechanisms of oocyte aneuploidy associated with advanced maternal age. Mutat. Res. Rev. Mutat. Res..

[33] Dakhno F.V., Hjort T., Grischenko V.I. (1980). Evaluation of immunofluorescence on pig zone pellucida for detection of anti-zone antibodies in human sera. J. Reprod. Immunol..

[34] Sacco A.G., Subramanian M.G., Yurewicz E.C. (1981). Passage of zona antibodies via placenta and milk following active immunization of female mice with porcine zonae pellucidae. J. Reprod. Immunol..

[35] Kamada M., Hasebe H., Irahara M., Kinoshita T., Naka O., Mori T. (1984). Detection of anti-zona pellucida activities in human sera by the passive hemagglutination reaction. Fertil. Steril..

[36] Buckshee K., Mhaskar A. (1985). Status of autoantibodies to zona pellucida in human reproduction. Int. J. Fertil..

[37] Karsten U., Donat H. (1986). Nachweis und Bedeutung von Zona-pellucida-Antikörpern im Serum steriler Frauen [Detection and significance of serum zona pellucida antibodies in sterile females]. Zentralbl. Gynakol..

[38] Gleicher N., Weghofer A., Kushnir V.A., Shohat-Tal A., Lazzaroni E., Lee H.J., Barad D.H. (2013). Is androgen production in association with immune system activation potential evidence for existence of a functional adrenal/ovarian autoimmune system in women?. Reprod. Biol. Endocrinol..

[39] Hasegawa A., Tanaka H., Shibahara H. (2013). Infertility and Immunocontraception based on zona pellucida. Reprod. Med. Biol..

[40] Conway G.S., Kaltsas G., Patel A., Davies M.C., Jacobs H.S. (1996). Characterization of idiopathic premature ovarian failure. Fertil. Steril..

[41] Calongos G., Hasegawa A., Komori S., Koyama K. (2009). Harmful effects of anti-zona pellucida antibodies in folliculogenesis, oogenesis, and fertilization. J. Reprod. Immunol..

[42] Weghofer A., Himaya E., Kushnir V.A., Barad D.H., Gleicher N. (2015). The impact of thyroid function and thyroid autoimmunity on embryo quality in women with low functional ovarian reserve: A case-control study. Reprod. Biol. Endocrinol..

[43] Polyzos N.P., Sakkas E., Vaiarelli A., Poppe K., Camus M., Tournaye H. (2015). Thyroid autoimmunity, hypothyroidism and ovarian reserve: A cross-sectional study of 5000 women based on age-specific AMH values. Hum. Reprod..

[44] Venables A., Wong W., Way M., Homer H.A. (2020). Thyroid autoimmunity and IVF/ICSI outcomes in euthyroid women: A systematic review and meta-analysis. Reprod. Biol. Endocrinol..

[45] So S., Yamaguchi W., Murabayashi N., Miyano N., Tawara F. (2019). Effect of moderately increased thyroid-stimulating hormone levels and presence of thyroid antibodies on pregnancy among infertile women. Reprod. Med. Biol..

[46] Zhang H., Qiu H., Liu Z., Wu Y., Liu W., Huang C. (2024). Subclinical/overt hypothyroidism may be associated with diminished ovarian reserve in infertile women independent of thyroid autoimmunity. Front. Endocrinol..

[47] Lu Y., Wu S., Zhang X., Zhang X., Li X., Tan J. (2025). Thyroid autoimmunity is strongly associated with embryo quality in infertile euthyroid women receiving IVF/ICSI, especially patients with TSH values above 2.5 uIU/mL and poor ovarian response. BMC Pregnancy Childbirth.

[48] Safarian G.K., Niauri D.A., Kogan I.Y., Bespalova O.N., Dzhemlikhanova L.K., Lesik E.A., Komarova E.M., Krikheli I.O., Obedkova K.V., Tkachenko N.N. (2023). Impact of Antithyroperoxidase Antibodies (Anti-TPO) on Ovarian Reserve and Early Embryo Development in Assisted Reproductive Technology Cycles. Int. J. Mol. Sci..

[49] Giménez-Barcons M., Casteràs A., Armengol Mdel P., Porta E., Correa P.A., Marín A., Pujol-Borrell R., Colobran R. (2014). Autoimmune predisposition in Down syndrome may result from a partial central tolerance failure due to insufficient intrathymic expression of AIRE and peripheral antigens. J. Immunol..

[50] Harris P.A., Taylor R., Thielke R., Payne J., Gonzalez N., Conde J.G. (2009). Research electronic data capture (REDCap)—A metadata-driven methodology and workflow process for providing translational research informatics support. J. Biomed. Inform..

[51] Liu X. (2012). Classification accuracy and cut point selection. Stat. Med..

[52] Nahm F.S. (2022). Receiver operating characteristic curve: Overview and practical use for clinicians. Korean J. Anesthesiol..

